# Prevalence and Correlates of Social Stigma Toward Diabetes: Results From a Nationwide- Survey in Singapore

**DOI:** 10.3389/fpsyg.2021.692573

**Published:** 2021-07-09

**Authors:** Mythily Subramaniam, Edimansyah Abdin, S. Bhuvaneswari, P. V. AshaRani, Fiona Devi, Kumarasan Roystonn, Peizhi Wang, Ellaisha Samari, Saleha Shafie, Janhavi Ajit Vaingankar, Rob M. van Dam, Eng Sing Lee, Chee Fang Sum, Siow Ann Chong

**Affiliations:** ^1^Research Division, Institute of Mental Health, Singapore, Singapore; ^2^Saw Swee Hock School of Public Health, National University of Singapore, Singapore, Singapore; ^3^Singhealth Polyclinics, Singapore, Singapore; ^4^Clinical Research Unit, National Healthcare Group Polyclinics, Singapore, Singapore; ^5^Lee Kong Chian School of Medicine, Nanyang Technological University, Singapore, Singapore; ^6^Admiralty Medical Centre, Khoo Teck Puat Hospital, Singapore, Singapore

**Keywords:** stigma, diabetes mellitus, survey, multi-ethnic, attitudes

## Abstract

**Aims:** To examine the extent of social stigma toward diabetes among Singapore's multi-ethnic general population and determine whether this differs across socio-demographic sub-groups.

**Methods:** Data for this study came from a nationwide cross-sectional study. A diabetes stigma questionnaire comprising Social Distance Scale and Negative Attitudes and Stereotyping Scale was administered to those respondents who had not been diagnosed with diabetes. Exploratory factor analysis was conducted to determine the dimensionality of the instruments and validated using confirmatory factor analysis. Multiple linear regression analysis was conducted to examine associations between socio-demographic factors and measures of diabetes stigma.

**Results:** In all, 2,895 participants were recruited from the general population giving a response rate of 66.2%. Factor analyses found that a one-factor model resulted in an acceptable fit for both stigma scales, which measured social distance and negative attitudes and stereotyping, respectively. Multiple linear regression analyses identified Indian ethnicity (vs. Chinese), higher personal income (≥SGD2000 vs. < SGD 2000) and having close friends or family members who had been diagnosed with diabetes to be significantly associated with lower social distance scores while those aged 50–64 years and those with secondary and vocational education (vs. degree and above) were significantly associated with higher social distance scores. Those with a personal income of SG$2,000–3,999 and SGD $6,000 and above, and those with close friends or family members diagnosed with diabetes were significantly associated with lower negative attitudes and stereotyping scores. In contrast those aged 35 years and above, those with primary education and below, and those of Malay ethnicity were significantly associated with higher negative attitudes and stereotyping scores.

**Conclusions:** The study found a relatively low level of stigma toward diabetes in the general population of Singapore, although some stigmatizing beliefs emerged. While greater knowledge of diabetes could reduce stigma, anti-stigma messaging should be incorporated into the “War on Diabetes” programme in Singapore.

## Introduction

Diabetes is a serious, chronic condition due to its high prevalence and adverse economic and social impact. The International Diabetes Federation estimates that nearly half a billion of the world's population lives with diabetes today. This figure is estimated to reach 700 million by 2045 at current incidence rates (Saeedi et al., 2019). Diabetes is among the top ten causes of death in adults globally and was estimated to cause 4.2 million deaths and cost USD760 billion in health expenditure in 2019 (Forouzanfar et al., 2016; International Diabetes Federation, 2019). Type 2 diabetes (T2DM) has reached epidemic proportions in many populations (Unnikrishnan et al., 2017) and comprises 90% of the total diabetic population worldwide.

The prevalence of diabetes in Singapore is among the highest within Southeast Asia, and among high-income countries, Singapore's national diabetes prevalence of 14.2% is second only to Germany (15.3%) (International Diabetes Federation, 2019). Pre-diabetes afflicts an estimated 15% of Singapore's population, a third of whom are estimated to develop diabetes within the next decade (Wong et al., 2003). The total economic cost of diabetes for the entire working-age population is expected to increase from US$787 million in 2010 to US$1,867 million in 2050 in Singapore (Png et al., 2016).

Singapore declared a “War on Diabetes” (WoD) in 2016 to mitigate the public health and economic impacts of diabetes, focusing on T2DM as it is a largely lifestyle-based (and preventable) condition. Since then, the Ministry of Health (MOH) has set in motion a whole-of-nation combat against diabetes by establishing the “Diabetes Prevention and Care Taskforce” and implementing programs and initiatives across schools, public spaces, organizations, and public health institutions (Ministry of Health Singapore, 2020). These include healthier meal options, free community workouts, and subsidies to encourage diabetes screening. Patients and healthcare providers have been empowered to manage the disease holistically and prevent diabetes-related complications (Ministry of Health Singapore, 2020). These efforts promote prevention, early detection, intervention, and better disease management by leveraging public education, stakeholder engagement, data analysis, and research (Ministry of Health Singapore, 2020). Despite the early indication of its success in the form of increased uptake of diabetes screening, the precise impact of the WoD on Singaporeans' outlook and practices is not yet known (Abu Baker, 2019).

The World Health Organization (WHO) defines stigma as a “mark of shame, disgrace or disapproval that results in an individual being rejected, discriminated against and excluded from participating in several different areas of society.” Health-related stigma commonly impacts individuals with chronic health conditions, and the individuals believe that others (who do not have the health condition) perceive a personal characteristic or attribute as deviant and display negative attitudes or discriminate against them (Link and Phelan, 2006). In the context of diabetes, these attributes include injecting insulin or testing one's blood sugars in public, dietary restrictions, and physical traits such as obesity (Browne et al., 2013). Persons with diabetes, fearing stigmatization may be less likely to use or not use therapies that may be visible to “others,” such as taking insulin injections or self-monitoring glucose levels (Ong et al., 2014). The commonly held view that diabetes is a disease that affects people who overindulge in sugary foods or are inactive (Abdoli et al., 2018; O'Keeffe et al., 2020) adds to shame and self-blame among those with diabetes. This may lead to people avoiding disclosure to friends, colleagues, and health care professionals because they fear judgment or blame (Browne et al., 2013; Olesen et al., 2017). Consequences of experiencing stigma for those with diabetes thus span across medical, psychological, and behavioral spheres and ultimately impact society as a whole through increased utilization of healthcare resources and the associated burden (Browne et al., 2013).

It has been argued that stigma should be considered a fundamental cause that is associated with health inequalities in a manner similar to socio-economic status and acknowledged as an important determinant of population health and the effectiveness of planned interventions (Hatzenbuehler et al., 2013; Meyer et al., 2020). However, stigma can oftentimes go unrecognized as people who do not have diabetes presume that it is not a stigmatized condition. In contrast, persons with diabetes perceive stigma and internalize it to varying extents (Schabert et al., 2013; Kato et al., 2016). Globally, a steadily growing body of research in this field has established the role of stigma in diabetes management and related behaviors. Despite compelling evidence that persons with diabetes perceive and experience stigma and suffer negative consequences (Liu et al., 2017; Kato et al., 2020), systematic research on diabetes stigma is lacking.

Against this backdrop, the current study examines the extent of social stigma toward diabetes among Singapore's general population and whether this differs for socio-demographic sub-groups. We used data from a nationwide study that examined the knowledge, attitudes, and practices regarding diabetes (AshaRani et al., 2020).

## Materials and Methods

### Setting and Study Design

Data for this study comes from a nationwide cross-sectional study conducted from February 2019 to September 2020 to determine the knowledge, attitudes, and practices regarding diabetes in the general population of Singapore. Inclusion criteria for participating in the study comprised Singapore citizens and permanent residents, those aged 18 and above; belonging to one of the four ethnic groups (Chinese, Malays, Indians, and Others); ability to understand and speak in English, Chinese, Malay, or Tamil; and living in Singapore at the time of the survey. All residents who were uncontactable due to incomplete or incorrect addresses, and those living outside the country during the survey period were excluded from the study. Those who were unable to understand the questionnaire due to cognitive difficulties as well as those with severe physical or mental disorders who were unable to answer the questionnaire on their own, were also excluded from the study. Data were collected through face-to-face interviewer-administered questionnaires via computer-assisted personal interviews (CAPI) using handheld tablets. Participants were asked to indicate their language of preference when informed consent was sought from them.

Approval for conducting the study was obtained from the Institute of Mental Health's Institutional Research Review Committee and the National Healthcare Group's Domain Specific Review Board (NHG DSRB Ref 2018/00430). Research related activities were initiated only after obtaining written informed consent from the participants. Parental consent was sought for those aged 18–20 years as the official age of majority in Singapore is 21 years and above.

### Sample Size Calculation and Sampling

The sample size estimates were produced by running statistical power calculations for binary proportions to determine what sample sizes are necessary overall and subgroups to produce a precise estimate with a margin of error ≤ 0.05. Based on data from an earlier study that examined knowledge of diabetes and risk factors (Wee et al., 2002), a target sample size of 3,000 was estimated to be adequate to determine the general knowledge of diabetes in the population. The study used a disproportionate stratified sampling design. The proportion of respondents in the Chinese, Malay, and Indian ethnic groups was set to ~30%, while the proportion of respondents in each age group was set around 20% to ensure that a sufficient sample size for these population subgroups could be achieved to improve the reliability of estimates. The margin of error for the overall prevalence estimate was 2.5%, while the margin of error for the subgroups defined by age and ethnic groups was between 4.5 and 5%. The sample was a respondent-level sample drawn from a national administrative database that maintains the records of all residents in Singapore, which are updated regularly. For the results to be representative of the Singapore population, all estimates were analyzed using survey weights to adjust for age and ethnicity post-stratification, oversampling, and non-response. The protocol of the study methodology has been published in an earlier article (AshaRani et al., 2020).

### Questionnaire

A structured questionnaire was used to obtain socio-demographic information, including age, gender, ethnicity, marital status, personal income, educational and employment status, and self-reported diagnosis of diabetes.

Additionally, participants were asked, “Have any of your close friends or family members been diagnosed with diabetes?” to establish close contact with a person with diabetes.

### Diabetes Stigma Questionnaire

The diabetes stigma questionnaire comprised two different scales; one of the scales assessed social distancing while the other assessed negative attitudes and stereotyping toward diabetes and those diagnosed with diabetes. The items assessing social distance were developed based on the social distancing scale by Link et al. (1999), while those on negative attitudes and stereotyping were based on a literature search (The Lancet Diabetes Endocrinology, 2018) and expert opinion. This questionnaire was administered only to those respondents who said that they had not been diagnosed with diabetes.

In all, 7 items assessed social distancing, which assessed the willingness of people without diabetes to socialize with someone with diabetes, work closely with them, marry someone in their family etc. (Item 1–7, **Table 2A**). The response options ranged from 1 to 4, from “definitely willing, probably willing, probably unwilling, to definitely unwilling” with higher scores reflecting the desire to distance themselves socially from someone with diabetes. Similarly, 7 items assessed negative attitudes and stereotyping toward diabetes and people with diabetes (Item 1–7, **Table 2B**). The response options ranged from 1 to 5, from “strongly agree, agree, neither agree nor disagree, disagree, to strongly disagree” with the “strongly disagree” option reflecting lower negative attitudes. The items were reverse-scored, and thus, higher negative attitudes and stereotyping scores indicate higher diabetes stigma.

### Statistical Analyses

Descriptive statistics were calculated, including frequencies, percentages, means, and standard deviations of each item and the total scores across factors. A split-half sample was generated to obtain the underlying factors of the two stigma scales, and the 2,440 participants were randomly divided into two groups. Exploratory factor analysis (EFA) was conducted to determine the dimensionality of the scales on the first subsample (*n* = 1,220) to extract the factors. The derived factors from the EFA were then applied to the second subsample (*n* = 1,220) and validated using confirmatory factor analysis (CFA). EFA examined the polychoric correlations with robust unweighted least squares (ULSMV) estimator. A Crawford-Ferguson equamax (CF-EQUAMAX) rotation was then applied to obtain a more discriminating factor structure. Several criteria were used to determine the number of factors in EFA, such as eigenvalues (values >1.0), visual sighting of scree plot, identifying the pattern of loadings on each factor (i.e., loading > 0.30 or cross-loading items) (Brown, 2006), and robustness of interpretability for each solution. In CFA, the polychoric correlations with robust weighted least squares (WLSMV) estimator were used. The model fit of the final model was assessed using the following indices: comparative fit index (CFI), root mean square error of approximation (RMSEA), standardized root mean square residual (SRMR), and Tucker–Lewis Index (TLI). CFI and TLI values ≥ 0.95 are considered to be a reasonably good fit, while RMSEA values ≤ 0.06 and SRMR values ≤ 0.08 are considered to be acceptable fit (Hu and Bentler, 1999). Missing data were low across items (0.4–4.0%). Hence, a listwise deletion procedure was used in all analyses. Reliability in the context of the internal consistency of each subscale was evaluated using Cronbach's alpha coefficient. Multiple linear regression analysis was conducted to examine associations between socio-demographic factors and measures of diabetes stigma. Age, gender, ethnicity, education, marital status, employment, personal income, and having close friends or family members diagnosed with diabetes were included as predictor variables, while stigma scores were treated as dependent variables. All analyses were conducted using MPLUS and Stata 14.0.

## Results

In all, 2,895 participants were recruited from the general population (screened 5,698), giving a response rate of 66.2%. Of the 2,895 participants, 2,440 participants were included in the current analysis—after removing 455 respondents with diabetes. Table 1 presents the socio-demographic characteristics of the study participants (*n* = 2,440). The majority of participants were aged 18–49 (62.4%), ethnic Chinese (76.9%), married (59.7%), and employed (72.4%). Tables 2A,B shows the endorsement of items on the social distance and negative attitudes and stereotyping scale by the respondents. In terms of social distancing, 2.3% of the population stated that they were unwilling (probably unwilling or definitely unwilling) to be friends with someone with diabetes, 3.1% were unwilling to socialize with someone with diabetes, 12.6% were unwilling to employ someone with diabetes, and 28.2% were unwilling to have someone marry into their family with diabetes. In terms of negative attitudes and stereotyping, 6.5% agreed (agreed or strongly agreed) that they would avoid getting screened for diabetes as they did not want to know if they had diabetes, 19.1% agreed that those with diabetes are responsible for bringing this condition on themselves, 69.1% agreed that those with diabetes use the healthcare system more than an average person, and 81.3% agreed that having diabetes affects a person's health care insurance.

**Table 1:** Socio-demographic characteristics of the sample.

**Socio-demographic variables**	***N =* 2,440**	**Unweighted**	**Weighted**
**Age group**		%	%
18–34	809	33.2	32.8
35–49	661	27.1	29.6
50–64	589	24.1	24.6
65 and above	381	15.6	13.1
**Gender**			
Female	1,239	50.8	51.8
Male	1,201	49.2	48.2
**Ethnicity**			
Chinese	725	29.7	76.9
Malay	805	33.0	12.1
Indian	718	29.4	7.9
Others	192	7.9	3.0
**Education**			
Primary and Below	455	18.7	18.4
Secondary	548	22.5	19.7
Pre-University/Junior College	112	4.6	5.1
Vocational Training	237	9.7	6.8
Diploma	439	18.0	19.1
Degree, professional certification, and above	649	26.6	31.1
**Marital status**			
Single	704	28.9	31.9
Married/cohabiting	1,514	62.1	59.7
Divorced/separated	129	5.3	5.0
Widowed	92	3.8	3.32
**Employment**			
Employed	1,718	70.4	72.4
Economically inactive	612	25.1	23.9
Unemployed	110	4.5	3.8
**Monthly Personal Income (SGD)**			
Below 2,000	975	41.9	38.7
2,000 to 3,999	622	26.7	25.8
4,000 to 5,999	292	12.5	13.8
6,000 to 9,999	166	7.1	8.7
10,000 & above	103	4.4	6.1
No income	171	7.3	6.9

**Table 2A:** Frequency of endorsement of the items of the social distance scale.

**Social distance**	**Definitely**	**Probably**	**Probably**	**Definitely**
	**willing**	**willing**	**unwilling**	**unwilling**
	***n* (%)**	***n* (%)**	***n* (%)**	***n* (%)**
1. How willing would you be to socialize with someone who has diabetes?	1,692 (68.3)	680 (28.5)	54 (2.8)	9 (0.3)
2. How willing would you be to make friends with someone who has diabetes?	1,772 (72.0)	617 (25.7)	38 (1.8)	10 (0.5)
3. How willing would you be to have someone with diabetes start working closely with you on a job?	1,699 (68.8)	662 (27.9)	63 (2.7)	13 (0.6)
4. How willing would you be to have someone with diabetes marry into your family?	849 (30.2)	971 (41.6)	455 (21.8)	137 (6.4)
5. How willing would you be to employ someone with diabetes?	1,151 (45.7)	985 (41.7)	228 (10.1)	56 (2.5)
6. How willing would you be to travel in a taxi or bus driven by someone who has diabetes?	1,320 (53.9)	909 (37.0)	169 (7.4)	36 (1.7)
7. How willing would you be to forego the dessert if your close friend or family member who has diabetes has his/her meals with you regularly?	1,400 (61.7)	788 (30.8)	190 (5.8)	57 (1.8)

*% is a weighted percentage*.

**Table 2B:** Frequency of endorsement of the items of the negative attitudes and stereotyping scale.

**Negative attitudes and stereotyping**	**Strongly agree**	**Agree**	**Neither agree or disagree**	**Disagree**	**Strongly disagree**
	***n* (%)**	***n* (%)**	***n* (%)**	***n* (%)**	***n* (%)**
1. Those with diabetes are to be blamed for “bringing this condition on themselves”	74 (2.1)	480 (17.0)	395 (16.5)	1,079 (47.9)	406 (16.6)
2. Those with diabetes use the healthcare system more than an average person	159 (5.0)	1,500 (64.1)	365 (14.1)	325 (13.6)	68 (3.2)
3. I would not be comfortable if someone injected themselves with insulin in front of me	69 (1.7)	473 (19.0)	221 (8.7)	1,255 (54.6)	419 (16.0)
4. I avoid getting screened for diabetes (testing my blood sugar levels) as I don't want to know if I have diabetes	29 (1.1)	151 (5.4)	123 (4.8)	1,499 (63.5)	637 (25.3)
5. Having diabetes affects a person's healthcare insurance	302 (14.7)	1,481 (66.6)	259 (8.3)	257 (9.3)	36 (1.1)
6. Having diabetes would make it difficult for a person to retain their job	81 (2.7)	818 (32.0)	499 (20.8)	898 (39.9)	112 (4.6)
7. If I had diabetes I would find it very difficult to deal with it	103 (3.3)	897 (38.4)	393 (16.3)	903 (37.7)	129 (4.3)

*% is a weighted percentage*.

### Factor Extraction and Validation

#### Social Distance Scale

The plot of eigenvalues of the initial 7-item social distance scale indicated that a single factor solution was plausible. The range of standardized loadings in the social distance factor was 0.51–0.91. To confirm this factor structure, CFA of the one-factor model resulted in an acceptable fit. The model fit indices were as follows: CFI = 0.97, RMSEA = 0.08, SRMR = 0.04, and TLI = 0.96 (Table 3A).

**Table 3A:** Factor loadings and goodness-of-fit indices of the final model of the social distance scale.

	**Social distance**
**Question**	**Loadings**
How willing would you be to socialize with someone who has diabetes?	0.908
How willing would you be to make friends with someone who has diabetes?	0.892
How willing would you be to have someone with diabetes start working closely with you on a job?	0.862
How willing would you be to have someone with diabetes marry into your family?	0.645
How willing would you be to employ someone with diabetes?	0.766
How willing would you be to travel in a taxi or bus driven by someone who has diabetes?	0.655
How willing would you be to forego the dessert if your close friend or family member who has diabetes has his/her meals with you regularly?	0.506
**Goodness of fit indices of derived from CFA**	
Chi-Square Test of Model Fit	64.43
Degrees of Freedom	14
RMSEA	0.077
CFI	0.973
TLI	0.959
SRMR	0.041
Cronbach alpha	0.81

#### Negative Attitudes and Stereotyping Scale

The plot of eigenvalues for the initial 7-item scale indicated that one or two-factor solutions were plausible. Upon examination of each of the rotated solutions, including the pattern of factor loadings, i.e., cross-loading and loadings > 0.30, three items (“Those with diabetes use the healthcare system more than an average person,” “Having diabetes affects a person's healthcare insurance,” and “If I had diabetes, I would find it very difficult to deal with it”) were removed. A one-factor solution with four items was found to be optimal. The range of standardized loadings in the negative attitudes and stereotyping factor was 0.36–0.64, respectively. To confirm this factor structure, CFA of the one-factor model using the final four items resulted in an acceptable fit. The model fit indices were as follows: CFI = 0.98, RMSEA = 0.04, SRMR = 0.02, and TLI = 0.95. Factor loadings and goodness of fit indices of the final model are presented in Table 3B.

**Table 3B:** Factor loadings and goodness-of-fit indices of the final model of the negative attitudes and stereotyping scale.

	**Negative attitudes and stereotyping**
**Question**	**Loadings**
Those with diabetes are to be blamed for “bringing this condition on themselves”	0.37
Those with diabetes use the healthcare system more than an average person	–
I would not be comfortable if someone injected themselves with insulin in front of me in a social setting	0.64
I avoid getting screened for diabetes (testing my blood sugar levels) as I don't want to know if I have diabetes	0.60
Having diabetes affects a person's healthcare insurance	–
Having diabetes would make it difficult for a person to retain their job	0.36
If I had diabetes I would find it very difficult to deal with it	–
**Goodness of fit indices of derived from CFA**	
Chi-Square Test of Model Fit	4.078
Degrees of Freedom	2
RMSEA	0.041
CFI	0.984
TLI	0.951
SRMR	0.016
Cronbach alpha	0.49

Two separate diabetes stigma scores related to social distance and negative attitudes and stereotyping were generated by summing up the relevant items of the scales. The Cronbach's α coefficient for the social distance and negative attitudes and stereotyping scales were 0.81 and 0.49, respectively.

#### Socio-Demographic Determinants of Social Distance, Stereotyping, and Negative Attitudes Scales

Supplementary Table 1 shows the mean and standard deviation of the stigma scores (factor score) by socio-demographic factors. The mean (standard deviation) scores on the social distance, negative attitudes and stereotyping scales were 10.8 (3.3) and 9.5 (2.4), respectively. The Pearson's correlation coefficient between social distance and stereotyping and negative attitudes was positively significant (r = 0.36) (Supplementary Table 2).

Table 4 shows the determinants of the social distance and negative attitudes and stereotyping. Multiple linear regression analyses revealed that those of Indian ethnicity (vs. Chinese), with a higher personal income (≥SGD2000 vs. < SGD 2000) and having close friends or family members who had been diagnosed with diabetes were significantly associated with lower social distance scores while those aged 50–64 years (vs. those aged 18–34 years) and those with secondary and vocational education (vs. degree and above) were significantly associated with higher social distance scores.

**Table 4:** Socio-demographic determinants of social distancing and negative attitudes and stereotyping scores.

	**Social distance**			**Negative attitudes and stereotyping**		
**Sociodemographic variables**	Coef.^*^	**95% CI**	***p-*value**	Coef.^*^	**95% CI**	***p-*value**
**Age** (years)		Lower, Upper			Lower, Upper	
18–34	Ref.			Ref.		
35–49	0.4	−0.1, 0.9	0.158	0.6	0.2, 1.0	**0.008**
50–64	0.7	0.04, 1.4	**0.037**	0.6	0.1, 1.1	**0.013**
65 and above	0.7	−0.1, 1.5	0.089	0.6	0.01, 1.2	**0.045**
**Gender**						
Female	Ref.			Ref.		
Male	0.3	−0.1, 0.7	0.192	0.01	−0.3, 0.3	0.948
**Ethnicity**						
Chinese	Ref.			Ref.		
Malay	−0.2	−0.6, 0.2	0.235	0.4	0.1, 0.7	**0.005**
Indian	−0.5	−0.8, −0.1	**0.007**	0.2	−0.1, 0.5	0.116
Others	0.5	−0.1, 1.1	0.082	−0.1	−0.5, 0.3	0.708
**Education**						
Degree, professional certification, and above	Ref.			Ref.		
Primary and Below	0.5	−0.3, 1.4	0.197	0.9	0.3, 1.5	**0.002**
Secondary	0.9	0.3, 1.6	**0.007**	0.2	−0.3, 0.6	0.439
Pre-U/Junior College	0.2	−0.8, 1.1	0.738	−1.0	−1.6, −0.3	**0.002**
Vocational training	1.1	0.1, 2.0	**0.025**	0.4	−0.3, 1.1	0.236
Diploma	0.1	−0.4, 0.7	0.715	−0.1	−0.5, 0.4	0.792
**Marital status**						
Married/cohabiting	Ref.			Ref.		
Single	−0.4	−0.9, 0.1	0.085	0.04	−0.4, 0.4	0.862
Divorced/separated	−0.4	−1.2, 0.4	0.354	−0.7	−1.3,−0.1	**0.025**
Widowed	0.8	−0.8, 2.3	0.339	−0.1	−0.8, 0.6	0.748
**Employment**						
Employed	Ref.			Ref.		
Economically inactive	0.04	−0.5, 0.6	0.889	−0.1	−0.5, 0.3	0.480
Unemployed	−0.3	−1.2, 0.6	0.526	−0.1	−0.7, 0.6	0.771
**Monthly Personal Income (SGD)**						
Below 2,000 or no income	Ref.			Ref.		
2,000 to 3,999	−0.7	−1.2, −0.1	**0.023**	−0.5	−0.9,−0.1	**0.016**
4,000 to 5,999	−0.9	−1.6, −0.1	**0.024**	−0.4	−1.0, 0.1	0.098
6,000 to 9,999	−1.4	−2.2, −0.5	**0.002**	−0.7	−1.3,−0.1	**0.018**
10,000 & above	−1.9	−2.9, −0.9	**<0.001**	−1.2	−1.9,−0.5	**0.001**
**Close friend or family members with diabetes**						
No	Ref.			Ref.		
Yes	−1.1	−1.5, −0.6	**<0.001**	−0.5	−0.8,−0.2	**0.001**

* *regression coefficient (Coef.) was derived from multiple linear regression analysis after controlling for all covariates. p<0.05 shown in bold*.

Those with pre-university/junior college education (vs. degree and above), divorced/separated (vs. married/cohabiting), having a personal income of SG$2,000 to 3,999 and SGD $6,000 and above (vs. < SGD2000), and those with close friends or family members diagnosed with diabetes were significantly associated with lower negative attitudes and stereotyping scores. In contrast those aged 35 years and above (vs. those aged 18–35 years), those with primary education and below (vs. degree and above), and those of Malay ethnicity (vs. Chinese) were significantly associated with higher negative attitudes and stereotyping scores.

## Discussion

This is the first study on stigma toward diabetes in an Asian general population to the best of our knowledge. The item-wise endorsement of social distancing ranged from less than one in ten of the population being unwilling to be friends with someone with diabetes to about three in ten unwilling to have someone with diabetes marry into their family. This contrasts with the results from a previous study in Singapore that examined stigma toward those with mental illness using a similar methodology. The study found that three in ten of those who were presented with a vignette of a person with schizophrenia stated that they would be unwilling to be friends with the person, while about one in ten were unwilling to be friends with a person having depression. Almost eight in ten were unwilling to have someone with schizophrenia marry into their family, and six in ten felt similarly about someone with depression getting married into their family (Subramaniam et al., 2017).

The unwillingness to have someone with diabetes marry into the family has been reported in earlier studies among those with Type 1 diabetes. A qualitative study in Iran identified significant concerns about having a person with Type 1 diabetes as a potential spouse. These concerns included disease burden in the form of compliance to medication, hospitalization, and the risk of diabetes in offspring (Abdoli et al., 2013). A study of South Asians in the UK identified similar concerns among people with Type 1 and Type 2 diabetes (Singh et al., 2012). Younger adults with diabetes felt they were often pressured to hide their condition. The authors acknowledged that this trend could be related to the reluctance of community disclosure of any familial medical conditions as they are perceived to threaten marriage prospects. Participants may have had similar concerns about diabetes in the current study, although we did not specify the type of diabetes, and participants did not ask us about it. Participants may have been concerned about the ability of a person with diabetes to participate in family events, celebrations, and even religious fasting which is observed by ethnic Indian and Malay communities. They may also feel that the family would need to make changes in diet and lifestyle to accommodate a person with diabetes. In Singapore, where residents need to co-pay their healthcare costs, concerns about the cost of medication, hospitalizations, and diabetes-related complications requiring complex surgery may have added to the unwillingness. (Ministry of Health Singapore, 2018).

In terms of negative attitudes and stereotyping, only less than one in ten stated that they would avoid getting screened for diabetes as they did not want to know if they had diabetes and about two in ten agreed that those with diabetes are responsible for bringing this condition on themselves. However, nearly seven in ten agreed that those with diabetes use the healthcare system more than an average person. While the findings are reassuring in the sense that there was little “label avoidance” and very few participants' “blamed” people with diabetes and held them accountable for developing diabetes, a significant number felt that people with diabetes consume healthcare more than those without diabetes. Despite the wide range of medications available to treat diabetes, the healthcare cost of diabetes has increased over the years. This could be due to the increasing prevalence of diabetes and suboptimal glycemic control, leading to comorbidities and complications resulting in increased cost (Banerji and Dunn, 2013; Juarez et al., 2013; O'Neill et al., 2018).

Social stigma in the context of health is complex. It comprises five components—identifying and labeling differences, stereotyping those with the label, separating the stigmatized group (social distancing), discrimination and loss of status of the stigmatized group, and lastly, the exercise of power to exploit or control the stigmatized group (Link and Phelan, 2006). The factors of the diabetes stigma scales used in this study encompass the components of social distancing and labeling, and stereotyping. Discrimination, loss of status, and stigma power are components that should be measured from the perspective of a person with a health condition. Thus, they were not included in the current scale, which was administered to those without diabetes. While the scale was developed with the definition of social stigma in mind and fit indices and internal consistency of the two scales were acceptable, we acknowledge that this questionnaire is new and therefore should be tested further for reliability and validity.

Those aged 50–64 years (vs. those aged 18–34 years) expressed significantly more desire for social distancing toward those with diabetes. Similarly, those aged 35 years and above (vs. those aged 18–34 years) had higher negative attitudes and stereotyping toward diabetes and those with diabetes. It is possible that people belonging to younger age groups may have more access to diabetes-related information and hence may have better knowledge and understanding of diabetes. However, knowledge alone does not influence stigmatization. Those belonging to the younger age group may be more inclusive and open-minded due to exposure to diverse cultures, especially in educational and workplace settings (Janmaat and Keating, 2019).

Having lower education and income was significantly associated with higher scores on the social distancing as well as the negative attitudes and stereotyping scale. It has been suggested that those with higher education have more knowledge and a better understanding of illness. This knowledge could be explained by those more educated obtaining their diabetes-related information from a wider variety of sources such as books, online websites, and health professionals. Several studies suggest that knowledge can reduce stigma toward the disease and lead to more acceptance (Balfour et al., 2010; Subramaniam et al., 2020). It is also possible that those of higher socio-economic backgrounds have better access to healthcare and can afford diabetes treatment. Thus, they would probably not associate diabetes with poor outcomes like retinopathy and amputations that can significantly affect functioning. Perceiving diabetes as a condition that can be well-managed with few adverse outcomes may mitigate its stigma.

An important finding of the current study was that having close friends or family members with diabetes was significantly associated with lower diabetes stigma scores in both scales. These findings are in line with the contact hypothesis theory, which states that contact between two groups (in the current study those with and without diabetes), under certain conditions, can promote tolerance and reduce prejudice (Allport, 1954). However, work by Pettigrew and Tropp (Pettigrew and Tropp, 2006) suggests that even when Allport's conditions (equal status, common goals, etc.) are not met, and contact is unstructured, it can still reduce prejudice. Contact is suggested to work by diminishing negative affect such as fear and inducing positive affect such as empathy (Tausch and Hewstone, 2010), leading to acceptance and prejudice reduction. This finding has implications for future public messages and campaigns. Positive portrayals of people with diabetes, interactions with those with diabetes during routine activities, and friendships between people belonging to both groups can dispel the stigma and encourage inclusivity.

Participants of Indian ethnicity had significantly lower social distancing scores than those of Chinese ethnicity. Several factors may have contributed to the lower social distancing observed in the Indian ethnic group. Diabetes prevalence among ethnic Indians in Singapore is higher than for other ethnic groups (Health Promotion Board, 2011). Therefore, it is likely that people of Indian ethnicity had family members or friends with diabetes and were aware of their ability to participate in leisure and occupational activities despite having diabetes and thus did not discriminate against them. It is also possible that people of Indian ethnicity felt that they were “labeled” as a high-risk group for diabetes and therefore were more empathetic toward people with diabetes. However, our study found an association between Malay ethnicity and higher negative attitudes and stereotyping score, although those of Malay ethnicity also have a higher risk of diabetes as compared with those of Chinese ethnicity (Health Promotion Board, 2011). A study examining the control of cardiovascular risk factors among those with diabetes in the major ethnic groups in Malaysia found that the Malays had the worst achievement of target for most of the risk factors (Lee et al., 2013). This may place them at a higher risk of complications leading to more negative attitudes toward diabetes and those with diabetes in the community. While social distancing and negative attitudes and stereotyping scales were significantly positively related to each other, consistent with the stigma theory; we did not observe this relationship in the Indian or the Malay ethnic group. While social distancing was significantly lower among Indians, negative attitudes and stereotyping did not show a similar trend. Similarly, while Malays endorsed higher negative attitudes and stereotyping, social distance scores were not significantly higher. Further research especially the use of qualitative methodologies may lead to a deeper understanding of the attitudes.

Several limitations in this study must be acknowledged. Firstly, the study had a response rate of about 66%—largely due to the COVID-19 pandemic and the subsequent fears and social distancing measures restricting both participant recruitment as well as the willingness of people to do household surveys even before the “circuit breaker” period (a period where restrictions were placed on working on-site, traveling, and visiting to limit the spread of COVID-19 infection) was adopted in Singapore. While we adjusted for non-response bias, those refusing to do the study may be inherently different from those willing to participate, affecting our results. Social desirability bias could have impacted participants' self-reported responses to the social stigma instrument, given that social stigma, in general, has a negative connotation, and many of the items required explicit endorsement of one's rejection or unwillingness to associate with persons with diabetes. While the sample is multi-ethnic, the findings might not be generalizable to other Asian populations with its diversity of cultures, literacy, and economic development.

In conclusion, while our study found a relatively low level of stigma toward diabetes in the general population of Singapore, some stigmatizing beliefs emerged. The authors believe that while greater knowledge of diabetes could reduce stigma, anti-stigma messaging should be incorporated into the “War on Diabetes” programme in Singapore. In line with an integrated care perspective, stigma should be viewed as a crucial element of chronic disease management so that all the different stakeholders involved in a person with diabetes's care journey (i.e., healthcare professionals, caregivers, and employers) can be more mindful of their own stigmatizing attitudes or language. Multiple stakeholders should be involved in co-creating and implementing multi-level stigma reduction interventions, thereafter sustaining it through community engagement (Nyblade et al., 2019; Rao et al., 2019). Media portrayals should also be mindful of how reporting on diabetes is worded as it may otherwise induce stereotypes among readers and viewers. For example, awareness can also be built into media health promotion campaigns, whereby the public can understand that anyone is susceptible to diabetes, regardless of their current health status. This will help to reduce stigma by inculcating more “continuum beliefs” in the general Singaporean population. Such beliefs, as opposed to strictly categorizing others (such as normal vs. diabetic), have been found to reduce stigma (Helmus et al., 2019). Given that stigma is a complex construct with a myriad of contributing factors, a whole-of-society approach is required to minimize it as far as possible. These steps are vitally important to ensure that stigma does not contribute to distress and label avoidance among individuals with diabetes as well as those at a high risk of developing diabetes.

## Data Availability Statement

The raw data supporting the conclusions of this article will be made available by the authors, without undue reservation.

## Ethics Statement

The studies involving human participants were reviewed and approved by National Healthcare Group Domain Specific Review Board, Singapore. Written informed consent to participate in this study was provided by the participants' legal guardian/next of kin.

## Author Contributions

MS, EA, JV, RD, EL, CS, and SC contributed to the study conception and design. SC was the PI of the study and was awarded the funding. Questionnaires were developed by MS, JV, PA, ES, RD, EL, CS, and SC. Literature review was done by SB, MS, PW, KR, and FD. Translation of questionnaires, cognitive testing, interviewer training, data check, and field management was done by PA, SS, FD, PW, KR, ES, and MS. Data analysis was done by EA. The first draft of the manuscript was written by MS and all authors commented on previous versions of the manuscript. All authors read and approved the final manuscript.

## Conflict of Interest

The authors declare that the research was conducted in the absence of any commercial or financial relationships that could be construed as a potential conflict of interest.
