# Supplementary material for: Prevalence and Correlates of Social Stigma Toward Diabetes: Results From a Nationwide- Survey in Singapore
Source: Front Psychol. 2021 Jul 9;12:692573. doi: 10.3389/fpsyg.2021.692573 (PMC8298907; doi:10.3389/fpsyg.2021.692573)
Supplement: Supplementary file 1 [file Table_1.DOCX]

Supplementary Table 1 Mean and standard deviation of the stigma scale scores by sociodemographic factors.

| **Sociodemographic Information** |  |  |  |  |
| --- | --- | --- | --- | --- |
|  | Social Distance | | Stereotyping and Negative Attitudes | |
|  | Mean | SD | Mean | SD |
| **All** | 10.76 | 3.32 | 9.51 | 2.41 |
| **Age group in years** |  |  |  |  |
| 18-34 | 10.20 | 3.04 | 9.01 | 2.46 |
| 35-49 | 10.40 | 3.03 | 9.47 | 2.17 |
| 50-64 | 11.31 | 3.45 | 9.86 | 2.39 |
| 65 and above | 12.03 | 3.97 | 10.26 | 2.53 |
| **Gender** |  |  |  |  |
| Female | 10.72 | 3.28 | 9.55 | 2.39 |
| Male | 10.80 | 3.36 | 9.48 | 2.42 |
| **Ethnicity** |  |  |  |  |
| Chinese | 10.82 | 2.10 | 9.46 | 1.46 |
| Malay | 10.81 | 5.21 | 9.95 | 4.33 |
| Indian | 10.07 | 5.77 | 9.52 | 4.94 |
| Others | 10.83 | 5.30 | 9.13 | 3.57 |
| **Education** |  |  |  |  |
| Primary and below | 11.84 | 3.63 | 10.68 | 2.47 |
| Secondary School | 11.63 | 3.67 | 9.68 | 2.40 |
| Pre-U/Junior College | 10.68 | 2.90 | 8.42 | 1.97 |
| Vocational Institute/ITE | 11.30 | 4.28 | 9.76 | 3.21 |
| Diploma | 10.29 | 2.81 | 9.26 | 2.27 |
| Degree, professional certification, and above | 9.78 | 2.73 | 9.00 | 2.05 |
| **Marital status** |  |  |  |  |
| Single | 10.27 | 2.89 | 9.15 | 2.36 |
| Married/cohabiting | 10.94 | 3.42 | 9.70 | 2.36 |
| Divorced/separated | 10.83 | 3.15 | 9.19 | 2.60 |
| Widowed | 12.32 | 5.10 | 10.14 | 2.70 |
| **Employment** |  |  |  |  |
| Employed | 10.55 | 3.20 | 9.43 | 2.34 |
| Economically inactive | 11.41 | 3.62 | 9.75 | 2.57 |
| Unemployed | 10.82 | 3.32 | 9.71 | 2.53 |
| **Monthly Income in SGD** |  |  |  |  |
| Below 2,000 or no income | 11.53 | 3.74 | 9.96 | 2.56 |
| 2,000 to 3,999 | 10.56 | 3.05 | 9.33 | 2.42 |
| 4,000 to 5,999 | 10.25 | 3.02 | 9.31 | 2.19 |
| 6,000 to 9,999 | 9.83 | 2.54 | 9.07 | 1.91 |
| 10,000 & above | 9.30 | 2.22 | 8.61 | 1.79 |
|  |  |  |  |  |

ITE: Institute of Technical Education; SGD: Singapore Dollars

Supplementary Table 2. Correlation matrix between stigma scales

|  | Social Distance | Stereotyping and Negative Attitudes |
| --- | --- | --- |
| Social Distance | 1.00 |  |
| Stereotyping and Negative Attitudes | 0.36* | 1.00 |
|  |  |  |

* Significant Pearson's correlation coefficient
